# Adverse effects of *Microcystis aeruginosa* exudates on the filtration, digestion, and reproduction organs of benthic bivalve *Corbicula fluminea*

**DOI:** 10.1038/s41598-024-61333-7

**Published:** 2024-05-13

**Authors:** Zijin Hong, Xinyun Chen, Junxiang Hu, Xuexiu Chang, Yu Qian

**Affiliations:** 1 Yunan Key Laboratory for Plateau Mountain Ecology and Restoration of Degraded Environments, School of Ecology and Environmental Sciences, Yunnan University, Kunming, 650091 Yunnan China; 2 Great Lakes Institute for Environmental Research, University of Windsor, Windsor, ON N9B 3P4 Canada; 3 Yunnan Collaborative Innovation Center for Plateau Lake Ecology and Environmental Health, College of Agronomy and Life Sciences, Kunming University, Kunming, 650214 China

**Keywords:** Biochemistry, Ecology, Ecology, Environmental sciences

## Abstract

Cyanobacteria bloom and the secondary metabolites released by the microorganism are extremely harmful to aquatic animals, yet study on their adverse effects in zoobenthos is rare. *Corbicula fluminea* widely distributed in freshwater environment with algal blooms. It is a typical filter feeding zoobenthos that may be affected by the secondary metabolites of cyanobacteria due to its high filtering rate. In this study, *C. fluminea* was exposed to *Microcystis aeruginosa* exudates (MaE) for 96 h, which was obtained from 5 × 10^5^ cells/mL and 2.5 × 10^6^ cells/mL exponential stage *M. aeruginosa* culture solution that represented cyanobacteria cell density needs environmental risk precaution control and emergent control, respectively. The responses of *C. fluminea* critical organs to MaE were analyzed and evaluated based on histopathological sections, antitoxicity biomarkers, and organ function biomarkers. The results showed that all the organs underwent structural disorders, cell vacuolization, apoptosis, and necrosis, and the damage levels increased as MaE concentration increased. The detoxification and antioxidant defense systems biomarkers in each organ response to MaE exposure differently and the level of reaction improved when MaE concentration increased. The siphon rate and acetylcholinesterase activity showed that the filtration function decreased significantly as the MaE concentration increased. Increased activity of glutathione S-transferase and amylase in the digestive gland indicate that it is the major detoxification organ of *C. fluminea*. Increased vitellogenin concentration and enlarged oocytes in the gonad indicate that MaE may have an estrogenic effect on *C. fluminea*. This study demonstrates that cyanobacteria threat benthic bivalves by inducing oxidative stress, inhibiting filtering feeding system, and disturbing digestion system and reproduction potential of *C. fluminea*.

## Introduction

Cyanobacterial harmful algal blooms (cHABs) are attracting increasing attention because of their effect on aquatic organisms and human health^1,2^. *Microcystis aeruginosa* is one of the most frequently occurring cyanobacteria worldwide^2^. The biological hazards of cHABs are mainly caused by the various secondary metabolites they produce^1–3^. Most research on the secondary metabolites of cyanobacteria has mainly focused on phycotoxins (i.e., microcystin, gangliotoxin, cylindricin, etc.)^4,5^; the adverse effects of bioactive secondary metabolites secreted by cyanobacteria have not yet been well studied^3,6,8,9^. Taking *M. aeruginosa* exudates (MaE) as an example, metabolites other than phycotoxins were identified from the exudate of *M. aeruginosa* in logarithmic phase, including lipids, organic heterocyclic compounds, organic acids, benzene like compounds and organic oxygen compounds^6^. Recent studies on MaE suggest that cyanobacterial secondary metabolites may be more toxic to organisms than microcystin^7^. Several studies have found that MaE promotes the reproduction of *Daphnia magna*^8^ and is teratogenic, neurotoxic, immunotoxic, and reproductively toxic to fish^9–11^. Although which specific compound in MaE caused the adverse effects above is still under investigation^10^, the aquatic organisms are inescapably under the exposure of the secondary metabolites generated by *M. aeruginosa* in water bodies with the cyanobacteria bloom^12^. Therefore, it is necessary to investigate how the aquatic organisms responds to MaE exposure. The zoobenthos is an important part of aquatic ecosystem, yet studies focus on the ecotoxicological effects of MaE on zoobenthos are rare. To explore the ecotoxicological effects of cyanobacterial exudates on zoobenthos is of great significance for water environment protection.

Benthic bivalves are important components of zoobenthos community in the aquatic ecosystem because of their important ecological functions, such as water filtration, nutrient element circulation, and microorganism excretion^13–15^. *Corbicula fluminea* is a typical benthic bivalve found in freshwater environments. It is commonly used for aquatic studies because of its unique characteristics, such as small individual size, low mobility, wide distribution, and rapid response to stress^14,16–18^. The respiration, filter feeding, digestion, and reproduction functions of *C. fluminea* are rooted in its gill, mantle, digestive gland, and gonad^19,20^. Recently, studies have found that the density and biomass of *C. fluminea* populations were significantly reduced in waters with cyanobacteria outbreaks^21,22^, indicating that cyanobacteria secondary metabolites had adversely affected the organism. Although detoxification and antioxidant systems do not directly impact organ functions, they play a critical role in maintaining cellular homeostasis when an organism is exposed to environmentally toxic chemicals. When these two systems are unable to endure the toxicity of MaE, tissue damage and organ function failure may occur^23^. Currently, there are few studies on the adverse effects of MaE on *C. fluminea* organs and their functions^22^. Therefore, exploring the ecotoxicological effects of MaE on *C. fluminea*, in particular the response of their important organs to MaE exposure, is urgently required.

We hypothesized that MaE can affect the organs of *C. fluminea* and further disturb organ functions, and biomarkers can be used to evaluate the sensitivity of *C. fluminea* organs to MaE exposure. This study aimed to investigate the responses of *C. fluminea* organs to MaE toxicity and to evaluate how organ function is influenced by MaE toxicity.

## Materials and methods

### Laboratory domestication of* C. fluminea*

We collected mature *C. fluminea* (hermaphroditic, shell length 2.0 ± 0.2 cm and shell height 1.0 ± 0.5 cm) from the aquatic product market in Sichuan Province, China^24,25^. We placed the *C. fluminea* in a 40 × 30 cm^2^ semiclosed circulation system: 20 °C ± 1 °C, 14:10 h light:dark cycle, no less than 6 mg/L dissolved oxygen, pH 7.8 ± 0.2^26^. We fed *C. fluminea* with 0.5 g of inactivated Chlorella powder twice a day during an acclimatization period^26^. After acclimation for more than 10 days, healthy *C. fluminea* of the same size were used for the experiments.

### *M. aeruginosa* culture and extraction of cyanobacterial exudates

*M. aeruginosa* (FACHB-905) purchased from the freshwater algae bank of the China Academy of Sciences was cultured in a light incubator (25 °C, light conditions of 2500 Lux, 12:12 h day:night cycle). To ensure that the MaE would remove as much of the algal toxins released by algal cell death as possible, we selected *M. aeruginosa* in the logarithmic phase to extract the extracellular secretions^9^. Therefore, *M. aeruginosa* was counted in the algae liquid every day during culturing to ensure that the algae cells were in the logarithmic growth stage (growth rate > 0.3). To extract the MaE, a 0.22 um cellulose acetate membrane was used on an ultra-clean bench to filter the algae liquid using a suction filtration device, and the filtrate was retained for the experiments.

### Laboratory exposure experimental design

All experiments were performed in triplicate on *C. fluminea* control and treated groups. The control group (C) was treated with COMBO medium without MaE, the experimental treated group E1 was treated with 5 × 10^5^ cells/mL MaE, and the experimental treated group E2 was treated with 2.5 × 10^6^ cells/mL MaE. The MaE concentrations were selected according to the density of *M. aeruginosa* recorded in lakes with different degrees of cyanobacteria blooms^27–31^. According to WHO guidance^32^, E1 (5 × 10^5^ cells/mL) is the precaution cyanobacteria bloom cell density, above which potential ecological risk may be occur. E2 (2.5 × 10^6^ cells/mL) is the cyanobacteria bloom cell density with high environmental risk, above which emergent management actions should be taken to inhibit cyanobacteria growth.

Each experimental group included 30 *C. fluminea* in an 18 L semiclosed system. The survival space of *C. fluminea* in the treated groups was guaranteed to be greater than 5 ind./L solution and 10 cm^2^/ind. We set the exposure time to 96 h because *C. fluminea* has been shown to be ecologically significant as a potential rapid response indicator for the environmental monitoring of short-term (96 h) exposures^33^. During treatment, Chlorella powder was fed every 48 h to exclude potential interference of starvation on the response of *C. fluminea* to MaE exposure. By the end of exposure, 6 *C. fluminea* were randomly selected for biochemical indicator analysis, 3 individuals were used for siphon rate evaluation, and another 3 organisms were used for histological section observation.

### Siphon rate

The siphon rate of *C. fluminea* was measured using the change in the absorbance of the water body resulting from the loss of neutral red dye particles due to filtration by *C. fluminea*. At the end of the MaE exposure experiments, three *C. fluminea* from each experimental group were placed in a 300 mL beaker containing 100 mL of neutral red solution (1 mg·L^−1^ in distilled water) and allowed to siphon for 2 h. Prior to placing *C. fluminea* in this solution, a 1 mL aliquot of water was removed from each beaker, and the absorbance at 530 nm was measured using an ultraviolet spectrophotometer to determine the concentration of the neutral red dye, which was used to generate a standard curve. After the siphoning for 2 h, another water sample was removed, and the absorbance was measured in the same way. The following formula was used to calculate the filtration rate:

1 $$m=\left[\frac{M}{nt}\right]log\left(\frac{{C}_{0}}{{C}_{t}}\right)$$

where *M* is the volume of the test solution,* n* is the amount of *C. fluminea* used, *t* is time (h), *C*_*0*_ is the initial concentration of dye, *C*_*t*_ is the dye concentration at time t, and *m* is the filtration rate (mL/animal/h).

### Histological observations

*C. fluminea* digestive glands and gills from each experimental group were fixed with 4% formaldehyde at 4 °C for 48 h using traditional classical histological observation methods. After storage in 70% ethanol, the samples were dehydrated, embedded in resin, cut into 4.5 μm slices, stained with hematoxylin and eosin, and observed under an optical microscope (BX53; Olympus, Tokyo, Japan).

### Biochemical indicator measurement

We evaluated the oxidative damage and detoxification metabolism of *C. fluminea* after exposure to MaE by detecting the activity of cytochrome P450 (CYP450), glutathione S-transferase (GST), superoxide dismutase (SOD) and concentration of malondialdehyde (MDA) in the mantle, gill, digestive gland and gonad of *C. fluminea*; The activity of acetylcholinesterase (AChE) in mantle and gill were measured to investigate possible mechanism behind the changed respiratory rate of *C. fluminea*^34–36^. The effect of MaE on the reproductive function of *C. fluminea* was evaluated by detecting the concentration of vitellogenin (Vtg) in the gonad of *C. fluminea*^37,38^. The effect of MaE on digestive function of *C. fluminea* was evaluated based on the activity of amylase in digestive gland^39^. These parameters were tested according to detection kits applicable to benthos provided by Shanghai Hengyuan biology Limited company.

*C. fluminea* individuals from exposure experiment was rinsed with ultra-pure water, and the organs (i.e., mantle, gill, digestive gland, and gonad) were separated by dissection. Six samples were collected from each type of tissue. The tissues were then rinsed with precooled PBS (0.01 M, pH = 7.4). After weighing, the tissues were cut into pieces and mixed to minimize individual differences. Then, 1 g of the tissue was mixed with a corresponding volume of PBS (1:9 weight:volume) and milled in a mill tube for 1 min. The homogenate was then centrifuged for 10 min (centrifugal force 5000 *g*, temperature 4 °C), and the resulting supernatant was used for the activity and concentration detection of biochemical indicators. To prevent temperature from affecting the activity of the biochemical indicators, all the experiment operations above were conducted in an ice bath. Three parallel samples were taken from each experimental group.

### Statistical analysis

The raw experimental data were processed and normalized using Excel 2019 software, and all results are presented as the SEM ± means. Correlations between the *C. fluminea* SOD, MDA, GST, CYP450, AChE, and siphon rate data were statistically analyzed using SAS JMP Statistical 13.2 software. The T test was used to evaluate the significance of the differences between the treated groups and the control group at different concentrations, with the significance level taken as p < 0.05. To determine relationships across MaE toxicity, *C. fluminea* internal defense system activation, and organ function response, factor analysis was conducted. VARIMAX rotation was used to rotate the variable matrix in the principal component analysis to a simple orthogonal structure. All graphics were drawn using GraphPad Prism 8 (GraphPad Software, USA).

## Results

### Histopathological changes in *C. fluminea* affected by MaE

**Figure 1:**
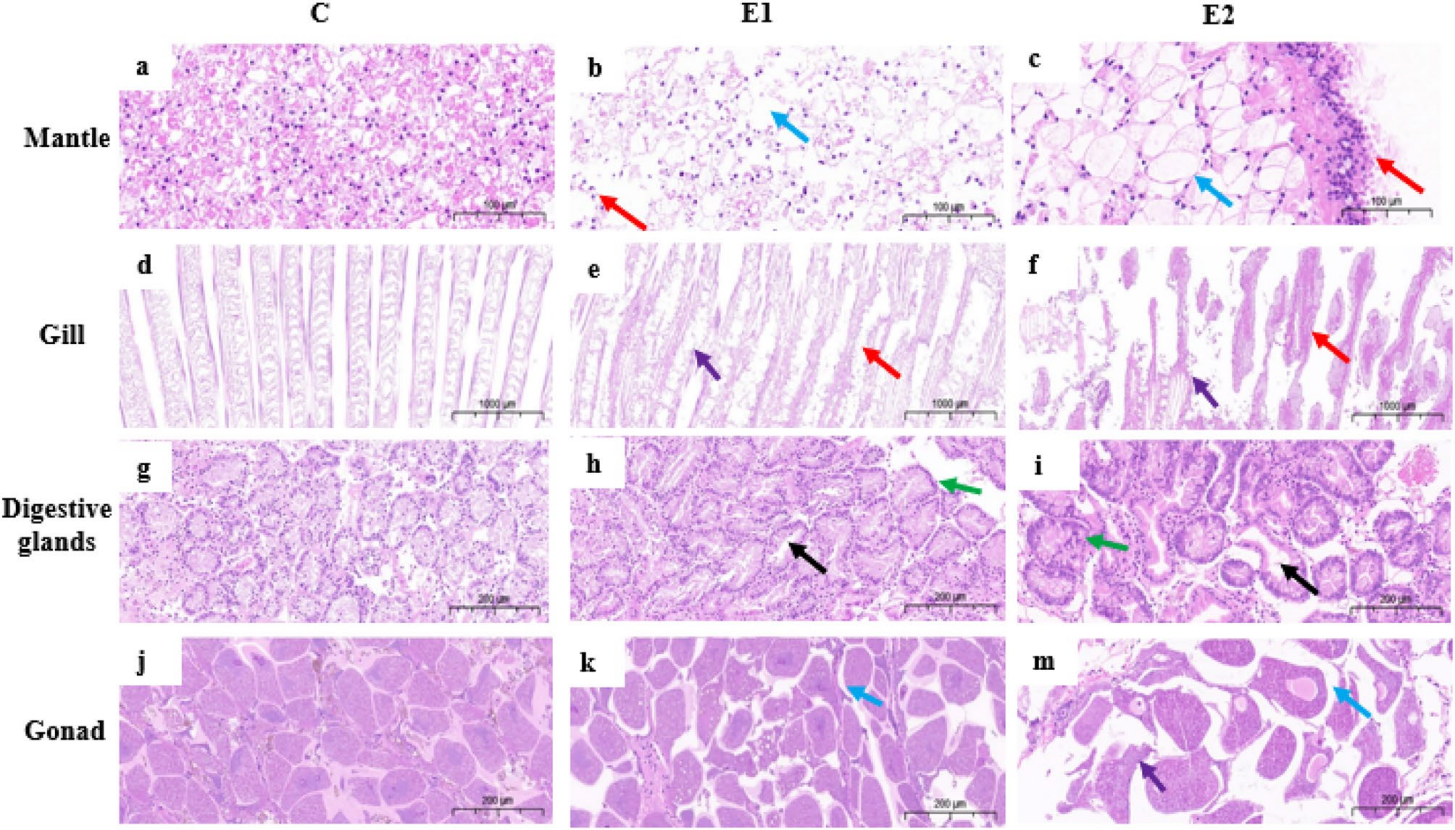
Histological changes in mantle (**a**–**c**), gill (**d**–**f**), digestive glands (**g**–**i**), gonad (**j**–**m**) of *C. fluminea* treated with MaE (C = 0 cell/mL, E1 = 5 × 10^5^ cell/mL, and E2 = 2.5 × 10^6^ cell/mL) for 96 h. Different arrow colors represent various histological damage of organ tissues (blue: cell vacuolation, red: cell necrosis, black: digestive tube deformity, green: epithelial cell thickening, purple: cell structure damage).

After 96 h of MaE treatment, we found significant histological changes in the gill, mantle, digestive gland, and gonad of the treated groups, and the changes gradually worsened with increased MaE concentration (Fig. 1). In this study, the MaE-treated *C. fluminea* mantle tissues showed structural disorders, such as uneven cytoplasm, nucleus extrusion to one side of the cytoplasm, membrane swelling, cell vacuolization, apoptosis, and necrosis (Fig. 1a–c). These disorders became more serious with increased MaE concentration (Fig. 1c). In the case of the gill, compared to the control group, where well-defined lamella were observed to make up skeletal rods that support a single layer of ciliated epithelial cells, we found that the MaE treated *C. fluminea* gill comprised a large number of cell apoptosis, necrosis, and lamellar deformations (Fig. 1d–f). Moreover, the gill tissue from the E2 treated group was almost completely damaged or deformed (Fig. 1f). A healthy digestive gland has regular Y-shaped digestive tubules and masked lumens formed by a single epithelial cell layer composed of basophils and digestive cells (Fig. 1g). After exposure to MaE, the digestive tubules were enlarged or malformed, and the epithelial cells were either reduced or enlarged (Fig. 1h). With an increase in MaE concentration, these impairments became more serious (Fig. 1i). In the case of gonad, an oogenic follicle normally consists of oocytes with basophilic cytoplasm and a nucleus composed of a prominent eosinophilic germinal vesicle and a nucleolus (Fig. 1j). With increasing MaE concentration, the gonad of *C. fluminea* exhibited damage, such as oocyte enlargement, decreased cytoplasm, cell vacuolization, and cellular structural damage (Fig. 1k,m).

### Response of *C. fluminea* antioxidant and detoxification indices to MaE

**Figure 2:**
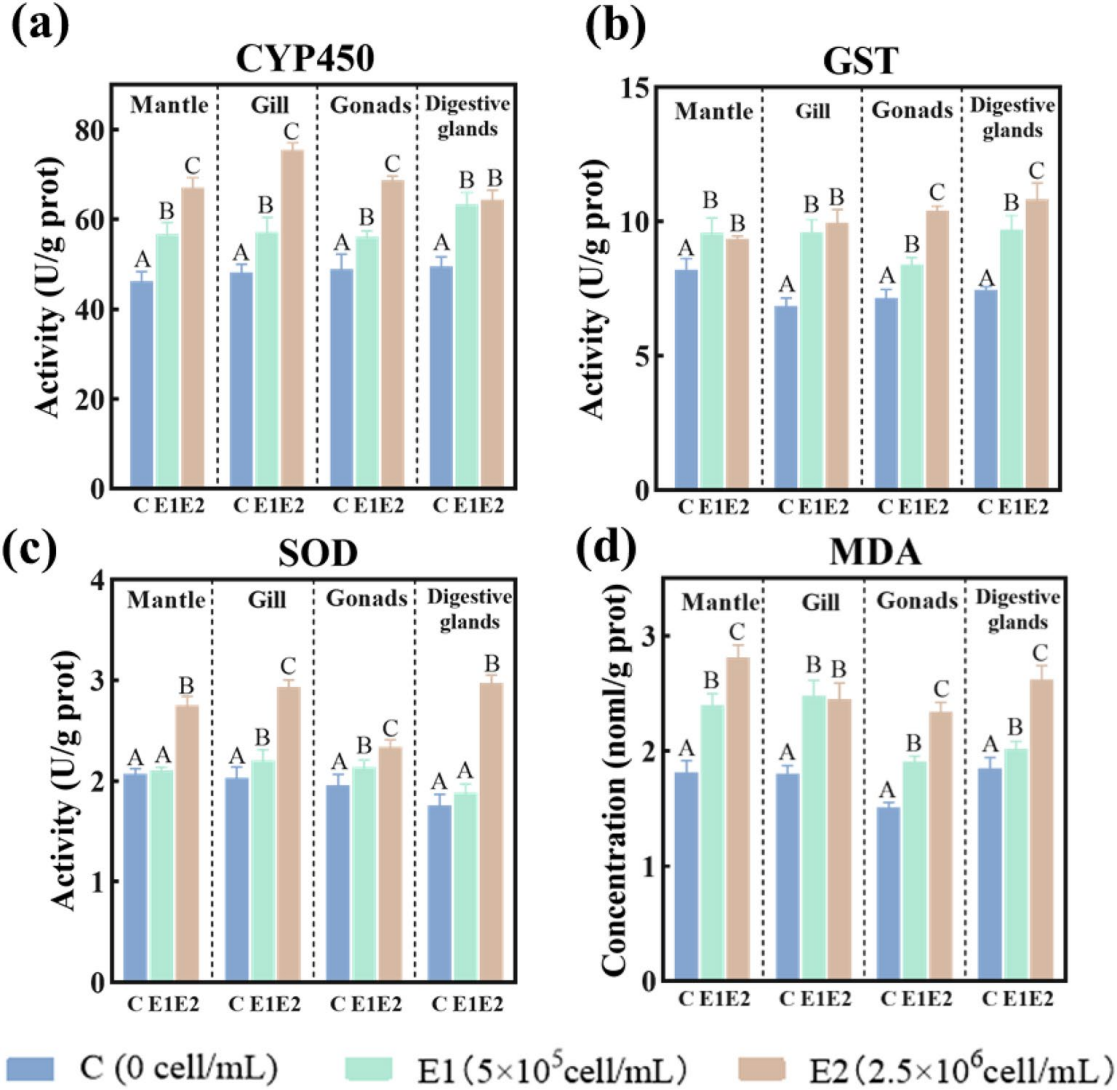
Activity changes of internal defense system enzymes ((**a**) phase I detoxification enzyme CYP 450; (**c**) phase II detoxification enzyme GST; (**c** antioxidant system enzyme SOD) and oxidative damage biomarker concentration ((**d**) MDA) in the mantle, gill, digestive glands and gonads of *C. fluminea* treated with MaE. All quantitative data are expressed as mean ± standard deviation (n = 3). The differences between the treatment group and the control group were analyzed and compared by significant differences, in which different letters in the figure show significant differences, *p* < 0.05).

CYP450 is a major monooxygenase in Phase I organic compound detoxification metabolic processes that oxidizes toxic organic compounds^40^. In this study, MaE treatment increased the CYP450 activity in the organs of *C. fluminea,* and the extent of the increase varied with organ type (Fig. 2a). The activity of CYP450 in the gill was the highest among all the organs in the E1 and E2 treated groups, and it was 19% and 56% higher, respectively, compared to the control group (Fig. 2a). GST is a Phase II detoxification enzyme that catalyzes the conjugation of glutathione to a variety of electrophilic functional groups on heterologous toxic organic compounds^34,41^. In this study, MaE treatment significantly increased the activity of GST in *C. fluminea*. The digestive gland had the highest GST activity after treated by MaE, followed by the gill, gonad, and mantle (Fig. 2b). Therefore, the digestive gland and gill may play critical roles in the Phase II detoxification of toxic compounds from MaE.

MaE treatment significantly impacted the oxidative stress defense system in the organs of *C. fluminea*, and the extent of the impaction varied with organ type and MaE concentration. SOD can transform reactive oxygen species (ROS) to H_2_O_2_^42^, and it is the most critical antioxidant against superoxide anion free radicals^43,44^. Generally, the activity of SOD increased in the mantle, gill, digestive gland, and gonad when MaE concentration went up. Although SOD activity in the mantle and digestive gland showed no significant increase in the E1 treated group, it significantly increased significantly (p < 0.05) compared to the control group in the E2 treated group. In the gill, the SOD active increased significantly (p < 0.05) by 9% and 44% in the E1 and E2 groups, respectively (Fig. 2c). SOD activity in E2 treated gill and gonad were higher than those observed in the mantle and digestive gland, implying that these two organs are the most affected by MaE. MDA level not only reflects the degree of cell membrane damage caused by oxidative stress but also causes biofilm degeneration and cell mutation or death^45,46^. Generally, the concentration of MDA in the investigated organs increased significantly (p < 0.05) with increased MaE treated concentration (Fig. 2d). MDA significantly increased in the E1 and E2 treated groups by 32% and 55%, respectively, in the mantle (p < 0.05); 38% and 36%, respectively, in the gill (p < 0.05); 9% and 42%, respectively, in the digestive gland (p < 0.05); and 26% and 55%, respectively, in the gonad (p < 0.05). Among the organs, the concentration of MDA in the treated mantle was noticeably higher than in other organs, indicating that cell membrane damage was most severe in the mantle, followed by the gill, digestive gland, and gonad (Fig. 2d). The changes of SOD activity and MDA concentration in different tissues of *C. fluminea* showed that the degree of oxidative damage to the mantle and gill was higher than that in the digestive gland and gonad.

### Effect of MaE on the organ function of *C. fluminea*

**Figure 3:**
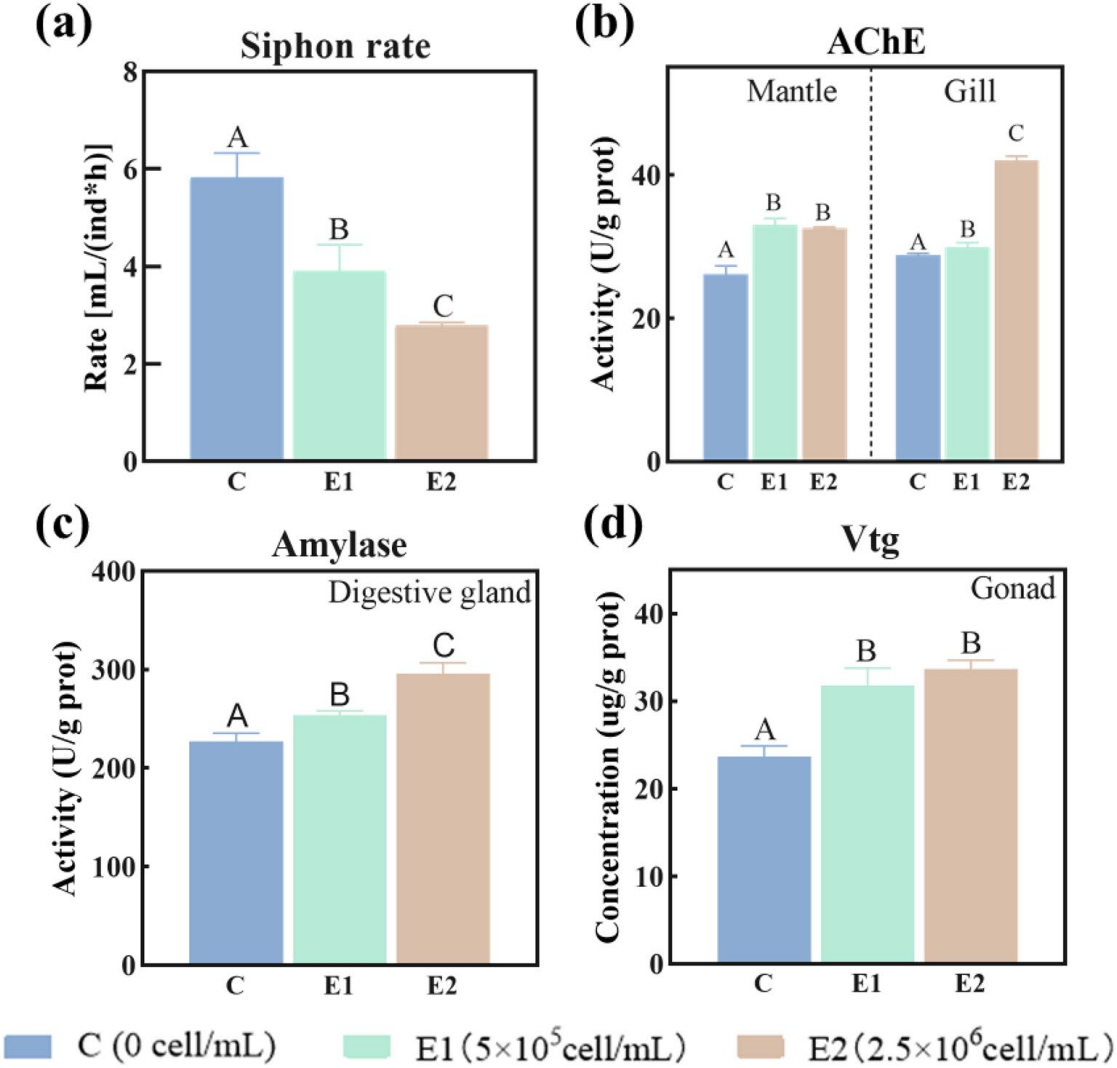
Changes in the organ function biomarkers of *C. fluminea* after treated with MaE. Respiratory and filter feeding biomarker, siphon rate (**a**) and AChE activity in mantle and gill (**b**); digestion function biomarker amylase activity in the digestive gland (**c**); reproduction function biomarker Vtg concentration in the gonad (**d**). All quantitative data are expressed as mean ± standard deviation (n = 3). The differences between the treatment group and the control group were analyzed and compared by significant differences, in which different letters in the figure show significant differences, *p* < 0.05).

The major function related to the gill and mantle of *C. fluminea* is the siphon rate, a movement related to AChE activity–controlled neuron signal transduction^47^. In this study, MaE treated inhibited the respiration and filter feeding of *C. fluminea*. The E1 and E2 treatments significantly (p < 0.05) reduced the siphoning rate of *C. fluminea* by 31% and 52%, respectively, compared to the control (Fig. 3a). MaE significantly increased the activity of AChE in the mantle and gill. In the mantle, the E1 and E2 treatments significantly (p < 0.05) increased AChE activity by 15%, compared to the control (Fig. 3b). In the gill, the E1 and E2 treatments significantly (p < 0.05) increased AChE activity by 5% and 35%, respectively, compared to the control (Fig. 3b). Elevated levels of AChE reduce interactions between neurotransmitters and acetylcholine receptors, which reduces the binding efficiency of the neurotransmitters to the receptors, ultimately leading to impaired neural function in organisms^48^. In addition, increased AChE activity can also induce apoptosis^47,49^, which may lead to the inhibition of the respiration and filter feeding functions of *C. fluminea*.

Amylase catalyzes starch hydrolysis in digestive organs and is considered an indicator of digestive function^50,51^. The E1 and E2 treated groups significantly (p < 0.05) increased amylase activity in the digestive glands of *C. fluminea* by 6% and 12%, respectively (Fig. 3c). This indicates that the digestive function of *C. fluminea* is stimulated by MaE exposure, which may result in energy supply promotion. This could be a strategy the clam adopted to improve its resistance to MaE toxicity.

Vtg is an important protein involved in the reproductive and developmental processes of oviparous animals^52,53^. Compared to the control group, the level of Vtg in the gonad collected from the E1 and E2 treated groups was significantly (p < 0.05) increased by 34% and 42%, respectively (Fig. 3d). Since Vtg can promote the growth of egg cells and assist sperm fertilization^52,53^, MaE exposure may improve the reproduction potential of *C. fluminea*^8,54^.

## Discussion

### Detoxification and antioxidation system responses to MaE toxicity

The adverse impact of pollutants on organs can be comprehensively determined by internal defense system activation level and chemical exposure degree. Detoxification system, an important part of the internal defense system that actively decomposes and removes toxic chemicals from organ tissues, consists Phase I (represented by CYP450 activity) and Phase II (represented by GST activity) reactions^34,55^. Antioxidant system is also a part of the internal defense system, which is activated when excess ROS generated due to toxic chemical exposure. MDA production represents the degree of cell membrane lipid peroxidation caused by oxidative stress^56^. The chemical exposure degree is usually determined by exposure pathways, accumulation rates, and elimination rate. An organ can be exposed to a toxin through either direct contact with a medium containing the toxin or indirectly via the circulation system^42,57^. When the toxicity of pollutants exceeded the tolerance capacity of the internal defense system, tissue damage that can be characterized by histological changes (i.e., inflammation, necrosis, etc.) will occur, and eventually organ function disorder will happen^45,46,58^. Therefore, *C. fluminea* organs response to the toxicity of MaE differently due to variation in their internal defense system and exposure pathways.

The gill and mantle have direct contact with MaE in water, yet their internal defense system respond to MaE toxicity differently. In the two organs, Fig. 2a,b shows that CYP450 was sensitive to MaE concentration change, while GST was only slightly upregulated compared to the control. This suggests that the Phase I reaction is the main detoxification reaction in the two organs. However, regarding the antioxidation enzyme SOD, its activity in the mantle only significantly increased in the E2 treated group, while that significantly increased in the E1 treated group in the gill (Fig. 2c). This indicates that the antioxidant system was more activated by MaE toxicity in the gill. Nevertheless, both detoxification and antioxidation systems did not reduce the ROS level in the two organs efficiently, since the MDA level increased continuously (Fig. 2d). Tissue sections of the mantle and gill also showed many cells underwent necrosis and apoptosis (Fig. 1a–f), which are caused by oxidative stress^34^. This is possibly because the main function of the mantle and gill is to secrete shell, control water inflow, and participate in respiratory function, its detoxification and antioxidation ability is limited compared to the visceral mass^59^. Notably, the MDA concentration in the gill increased dramatically and there was no significant difference in MDA concentration between the E1 and E2 treatments (Fig. 2: d), possibly under the E2 treatment, the gill tissue was too damaged to generate MDA^44^. Therefore, compared to the mantle, the gill suffered more damage from the toxicity of MaE, and the tissue sections of the gill supported this. As shown in Fig. 1; slides d–f, the tissue structure of the gill in the E2 treated group was seriously damaged (i.e., a high proportion of cells underwent apoptosis, necrosis, and lamellar deformation).

The digestive gland can be exposed to MaE not only directly via the digestion of particle sorbs MaE, but also indirectly via the circulatory^60,61^. The highest accumulation of toxin chemicals also tends to occur in the digestive gland^62,63^. Nevertheless, the activation level of SOD and CYP450 under MaE exposure were low, although large increases in MDA concentration and GST activity were observed (Fig. 2b,d). This shows that detoxification, especially the Phase II detoxification process, was sensitive to MaE toxicity. In addition, significant oxidative damage (i.e., enlarged or deformed digestive tubule lumen and decreased or enlarged epithelial cells) was observed only in the E2 treated group, suggests the digestive gland is not significantly damaged by MaE toxicity. This can also be demonstrated in the tissue sections of the digestive gland (Fig. 1h–j). Possibly the relatively low MaE exposure level and the active detoxification process in the digestive gland tissue contributed to attenuation of MaE toxic effect.

The gonad can only be exposed to MaE via the circulatory system indirectly^60,61^. Therefore, the reaction of detoxification and antioxidant system in gonad to MaE exposure was weaker than that in the other organs. As shown in Fig. 2, the values of SOD and GST in the gonad was lower than that in the mantle, gill, and digestive gland, suggests limited activation of internal defense system^62,64^. In the case of MDA concentration, it increased continuously as the MaE concentration increased (Fig. 2d), although its value is lower than that in other organs. This implies that MaE still induced oxidative stress in the gonad tissue, yet its toxic impact is lower in the organ. The tissue sections confirmed MaE posed lower stress in the gonad, since only cell vacuolation and cellular structural damage was observed in the MaE treated gonad cells (Fig. 1, j–m).

### Impact of MaE on organ functions

MaE toxicity not only disturbed the detoxification and antioxidation system of the organs of *C. fluminea* and caused histological changes in the organ tissues, but also impacted the critical function of the organs. To investigate how MaE toxicity affects the organ functions, factor analysis was conducted to explore the internal relationship among detoxification system activity (GST and CYP450), antioxidant system activity (SOD), oxidative stress (MDA), and organ function (siphon rate, AChE, amylase, VTG) (Table 1).

**Table 1:** Results of factor analysis based on loading matrix of detoxification system, antioxidant system, oxidative stress, and organ function properties in the mantle, gill, digestive gland and gonad.

Mantle			Gill			Digestive gland			Gonad		
Parameter	Factor 1	Factor 2	Parameter	Factor 1	Factor 2	Parameter	Factor 1	Factor 2	Parameter	Factor 1	Factor 2
SOD	**0.85**	− 0.11	SOD	**0.89**	0.13	SOD	− 0.05	**0.98**	SOD	0.13	**0.97**
MDA	**0.89**	0.42	MDA	0.22	**0.97**	MDA	0.37	**0.90**	MDA	**0.93**	0.28
CYP450	**0.86**	0.45	CYP450	**0.90**	0.34	CYP450	**0.98**	− 0.11	CYP450	**0.89**	0.11
GST	0.24	**0.94**	GST	0.36	**0.93**	GST	**0.92**	0.26	GST	**0.94**	0.26
AChE	− 0.09	0.90	AChE	**0.88**	0.32	Amylase	**0.91**	0.28	Vtg	**0.68**	**0.69**
Siphon rate	** − 0.96**	0.06	Siphon rate	** − 0.92**	− 0.35						
Total variance explained	54.40%	34.90%		57.00%	36.00%		53.30%	38.30%		60.30%	31.50%

Numbers set in bold indicates the high loading value of the parameter.

The respiratory and filter feeding function, which can be represented by siphon rate, reduced significantly with increased MaE exposure concentration. The mantle of *C. fluminea* can influence siphon rate by controlling water circulation through body of the bivalve while movement of cilia on the gill regulates water flow^65^. Although reduced siphon rate represents organ dysfunction of the mantle and gill, the reason of organ disfunction is different between the two organs, as indicated by the factor analysis (Table 1). In the mantle, SOD, MDA, CYP450 and siphon rate shares high loading in Factor 1, while GST and AchE showed significant (p < 0.05) positive correlation in Factor 2. This indicates the negative contribution of mantle to siphon rate reduction is possibly related to cell membrane damage^66^ and the activation of the antioxidant and detoxification system^67^. Interestingly, high positive significant (p < 0.05) correlation is observed between GST and AchE, indicates the two enzymes involve in the detoxification process in the mantle of *C. fluminea*, which is usually observed in insects^68^. In the gill, however, siphon rate negatively correlated significantly (p < 0.05) with SOD, CYP450, and AchE in Factor 1, while GST and MDA shared high loading in Factor 2. It is worth to notice the significant negative correlation between siphon rate and AchE level indicated the decreased activity of AchE in the gill resulted in the inhibition of respiratory and filter feeding movement of *C. fluminea*. This is because increased AChE activity can increase the hydrolysis of neurotransmitter acetylcholine level, thus further reduce neural information transmission^69^ and inhibit the respiratory and filter feeding functions of *C. fluminea*^56^. Therefore, in this study, MaE not only disturbed the internal anti-oxidation system balance and induced the detoxification system in the mantle and gill, but also interfered with the normal nerve signal transduction in the gill. As the result, the respiratory and filter feeding function of *C. fluminea* is inhibited.

The digestive function represented by the activity of amylase in the digestive gland can be influenced by the toxicity of MaE. As showed in the factor analysis, CYP450, GST and amylase shared high loading in Factor 1, while SOD and MDA shared high loading in Factor 2 (Table 1). It can be deduced from the internal relationship showed above that although the digestion function of *C. fluminea* was not influenced by the oxidative stress caused by MaE toxicity, it was highly related to the detoxification activity in the digestive gland. The digestive gland is the main detoxification organ of *C. fluminea*^41,70^, activation of CYP450 and GST in the organ after MaE exposure suggested organic compound detoxification system was stimulated by MaE treatment. Although the function of amylase is catalyzing starch hydrolysis and providing energy, its significant positive correlation with CYP450/GST suggests *C. fluminea* may increase its resistance to MaE toxicity by supporting the detoxification system through promoting the digestion function^71–73^.

Vtg in the gonad can be considered as the indicator of reproduction function of *C. fluminea* because it can promote the growth of egg cells and assist sperm fertilization^52,53^. The reaction of gonad to MaE exposure differed from that of the other organs. As shown in Table 1, although Vtg shares high loading with MDA, CYP450, GST, and SOD in Factor 1 and Factor 2, the loading value was below 0.75, indicating moderate positive significant (p < 0.05) correlation between Vtg activation the internal defense system in the gonad. It is likely the promoted reproduction function by MaE is not directly related to the oxidative stress and internal defense system. Previous study suggested MaE may contain estrogen analogues and increase *D. magna* reproduction rate by stimulating the activity of 17 β- Hydroxysteroid dehydrogenase (17 β- HSD) and promoting the production of ecdysone and juvenile hormone^8^. Moreover, considering the activation level of the detoxification and antioxidant system in the gonad after MaE exposure was not as strong as that in other organs, the major impact of MaE on the gonad is stimulation of reproduction function.

More and more evidences indicate MaE can not only induce oxidative stress, but also adversely affect the nervous system, immune system, reproduction system, and embryonic development of aquatic organism^8,9,11,74^. The complex component of MaE might explain the integrated toxicity of MaE. At present, more than 2000 chemicals other than phycotoxins were identified from the secondary metabolites of *M. aeruginosa*, including lipids, organic heterocyclic compounds, organic acids, benzene-like compounds, and organic oxygen compounds^8,9,11^. Although several chemicals have been screened as potential reason of neurotoxicity (such as phytosphingosine, egonol glucoside, and dihydrosuberenol)^75^, reproductive interference (such as 8-iso-15-keto-PGE2, ricinoleic acid, and oleic acid), immunotoxicity (such as shinflavanone, stearidonic acid, and linoleamide)^10^, and cytotoxicity (phytosphingosine)^76,77^ caused by MaE, the toxic effect of MaE on aquatic organisms is the result of integrated toxicity of all the secondary metabolites. Therefore, it is still necessary to focus on the integrated ecotoxicological effects of extracellular secretions of cyanobacteria on organisms, especially which system and organ function of the organism is influenced the most^12^. In this study, the impact posed by MaE on *C. fluminea* includes oxidative stress, neurotoxicity, reproductive interference and digestion system stimulation. The respiratory and filter feeding function, the digestion function, and the reproductive function of *C. fluminea* are all influenced by MaE toxicity.

## Conclusion

This study found that after exposure to logarithmic stage MaE, critical *C. fluminea* organs responsible for respiration, filtration, digestion, and reproduction were adversely affected. At the tissue level, all the organ tissues showed histopathological damage, which increased with increased MaE concentration. Damage to the mantle and gill was worse than damage to the digestive gland and gonad. Regarding the detoxification and antioxidant defense systems, the gill and mantle are the most affected organs under MaE exposure, while the gonad and digestive gland are less impacted due to induced detoxification system and less MaE exposure degree. For organ function biomarkers, a reduction in siphon rate is an ideal indicator of damage caused by MaE in the gill and mantle, while increased Vtg concentration can be used to indicate the impact of MaE on the reproduction system of *C. fluminea*. In conclusion, freshwater cHABs can influence the physiological processes of bivalves. Physiochemical indices, such as GST, SOD, and MDA, and functional indices, such as siphon rate, amylase, and Vtg, have been shown to be ideal candidates for cyanobacteria bloom monitoring. The results of this study will provide valuable support for understanding the toxicology of cyanobacteria secondary metabolites on benthic organism.

## Data Availability

All data generated or analyzed during this study are included in this article. The original datasets used and/or analyzed during the study are available from the corresponding author on reasonable request.
